# GAD65 Antibody-Associated Epilepsy

**DOI:** 10.3390/medicina59061135

**Published:** 2023-06-12

**Authors:** Justina Valinčiūtė, Neringa Jucevičiūtė, Renata Balnytė, Giedrė Jurkevičienė, Giedrė Gelžinienė

**Affiliations:** 1 Faculty of Medicine, Medical Academy, Lithuanian University of Health Sciences, 44307 Kaunas, Lithuania; 2 Department of Neurology, Medical Academy, Lithuanian University of Health Sciences, 44307 Kaunas, Lithuania; neringa.juceviciute@gmail.com (N.J.); renatabalnyte@lsmu.lt (R.B.); giedre.jurkeviciene@lsmu.lt (G.J.); giedre.gelziniene@lsmu.lt (G.G.)

**Keywords:** autoimmune epilepsy, glutamic acid decarboxylase (GAD), autoimmune encephalitis, seizures

## Abstract

Autoimmune processes are an increasingly recognized cause of seizures. Antibodies against neuronal surface antigens are implicated in the development of acute symptomatic seizures secondary to autoimmune encephalitis, whereas antibodies against intracellular antigens (anti-glutamic acid decarboxylase (GAD) and onconeural antibodies) are found in cases of autoimmune-associated epilepsy (AAE). AAE is described as isolated drug-resistant epilepsy without any specific magnetic resonance imaging (MRI) or cerebrospinal fluid changes and with a very limited response to immunotherapy. We present a clinical case and a literature review on autoimmune-associated epilepsy to increase awareness of this disease and illustrate its complexity. This is a clinical case of a female with a history of refractory focal epilepsy. The patient had been given several trials of multiple antiepileptic drugs and their combinations without any clear effect. Multiple evaluations including brain MRI, PET, and interictal and ictal electroencephalograms were performed. An APE2 score was calculated with a result of 4 and, in the presence of anti-GAD65 antibodies in the serum, the diagnosis of AAE was confirmed. There was no effect after five sessions of plasma exchange; however, after a course of intravenous immunoglobulin, a positive but temporary clinical effect was noticed: anti-GAD65 levels initially decreased but rebounded to previous levels 6 months later.

## 1. Introduction

Epilepsy is one of the most common neurological disorders encountered in clinical practice with an estimated global prevalence of 52.5 million [1]. The possibility that some epilepsies without any apparent cause may be caused by autoimmune processes was raised in 2002 when the term autoimmune epilepsy was coined for the first time [2]. Since then, there has been a growing body of evidence linking immune mechanisms with the development of epilepsy, leading the International League Against Epilepsy (ILAE) to introduce the category of immune etiology in its classification system of the epilepsies alongside structural, genetic, infectious, metabolic, and unknown etiologies in 2017 [3]. Since its introduction, the term autoimmune epilepsy has been used to describe various conditions ranging from drug-resistant autoantibody-positive epilepsy without any evidence of ongoing encephalitis to the development of acute seizures in patients diagnosed with autoimmune encephalitis (AE). However, this ambiguous terminology led to a debate among experts whether seizures in the context of AE are truly in line with the definition of epilepsy since epilepsy is characterized by an enduring predisposition to generate epileptic seizures [4], whereas in the majority of autoimmune encephalitis cases, complete seizure freedom is achieved after immunomodulatory treatment [5]. For example, in a follow-up study of 88 patients who had seizures secondary to anti-N-methyl-d-aspartate receptor (anti-NMDAR) encephalitis, all patients achieved complete seizure freedom within two years, and the majority of them (>80%) had their last seizure within six months after disease onset [6]. Following this debate, in 2020, the ILAE discouraged the use of the term autoimmune epilepsy and, aiming to clearly define epileptic and symptomatic seizures of autoimmune etiology, suggested two new definitions: acute symptomatic seizures secondary to autoimmune encephalitis and autoimmune-associated epilepsy (AAE) [5]. We present a clinical case and a literature review on autoimmune-associated epilepsy in order to increase awareness of this disease and to illustrate its complexity.

## 2. Case Report

A female with a 7-year history of refractory focal epilepsy was referred to the Department of Neurology of Lithuanian University of Health Sciences Kaunas Clinics in 2020. The patient did not have any other remarkable medical history except for generalized anxiety disorder and insomnia for which she was taking quetiapine. She had a family history of diabetes but her own previous glucose tolerance results were normal. At the time the patient was first seen in our department, she had focal impaired awareness seizures with a variable frequency ranging from 1 to 15 per day as well as nocturnal focal to bilateral tonic–clonic seizures occurring once every 1–2 months. The patient described the former type of seizures as ~1 min episodes of nausea, hypersalivation, and “weird” sensations in the epigastric region; during these episodes, she can hear other people talking but cannot reply or determine what they were saying. These episodes began about 13 years ago, in 2007, with very low frequency and were attributed to an anxiety disorder, and it was not until 2013 that the diagnosis of epilepsy was made after recording focal epileptiform activity for the first time on an electroencephalogram (EEG). Because the patient had been followed up in another hospital, not all previous medical records were available to us, but to the best of our knowledge, at the time of epilepsy diagnosis, both routine blood testing and the first brain MRI were unremarkable.

Since 2013, the patient had been given several trials of multiple anti-epileptic drugs (AEDs) and their combinations, most of which were discontinued due to inefficacy or side-effects (carbamazepine, oxcarbazepine, valproic acid, lamotrigine, pregabalin, gabapentin, topiramate, zonisamide, and various benzodiazepines). Since 2018, she has been treated with levetiracetam 2000 mg per day, which was partly effective at reducing by half the frequency of nocturnal focal to bilateral tonic–clonic seizures. The patient did not report any increase in her anxiety when being treated with levetiracetam In 2019, the hypothesis of autoimmune-associated epilepsy for the first time was raised, and the patient underwent testing for antibodies with the revelation of an anti-glutamic acid decarboxylase 65 (GAD65) titer of <1:20 in the cerebrospinal fluid (CSF) and serum. However, at that time, it was decided that there were not enough data to support the diagnosis of autoimmune-associated epilepsy, and the possibility of other systemic autoimmune disorders was recommended to exclude. 

**Figure 1:**
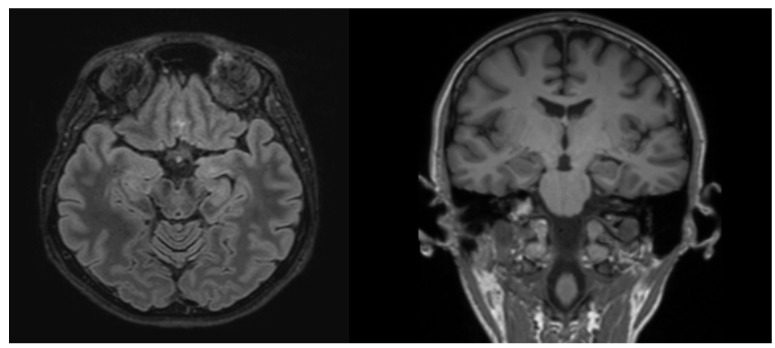
Brain MRI (3T) axial T2-FLAIR (**left**) showing a small gliosis area (~2.5 mm) in the left amygdala and coronal T1 (**right**) slight hippocampal asymmetry due to incomplete left hippocampal inversion.

**Figure 2:**
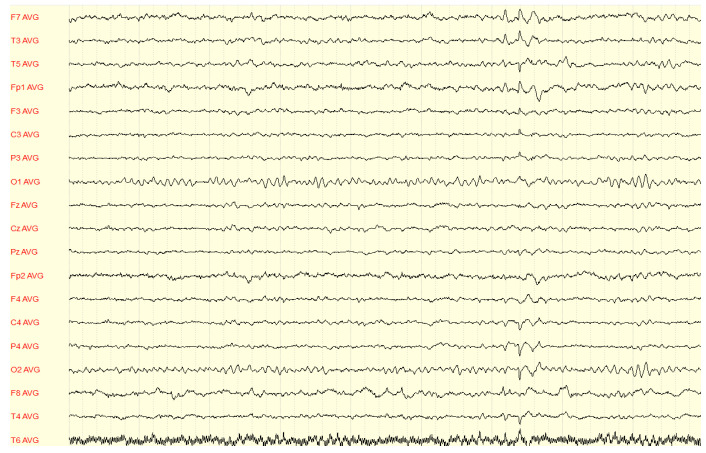
EEG findings during the interictal phase: left frontotemporal epileptiform discharges.

**Figure 3:**
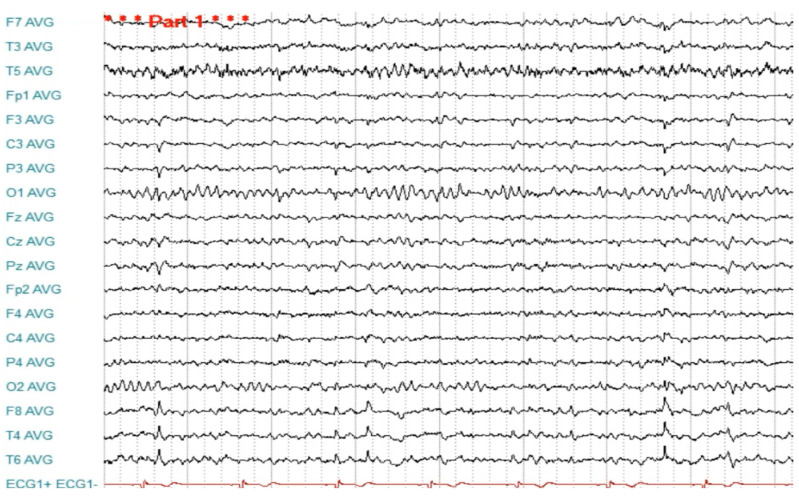
EEG findings during the interictal phase: right frontotemporal epileptiform discharges.

**Figure 4:**
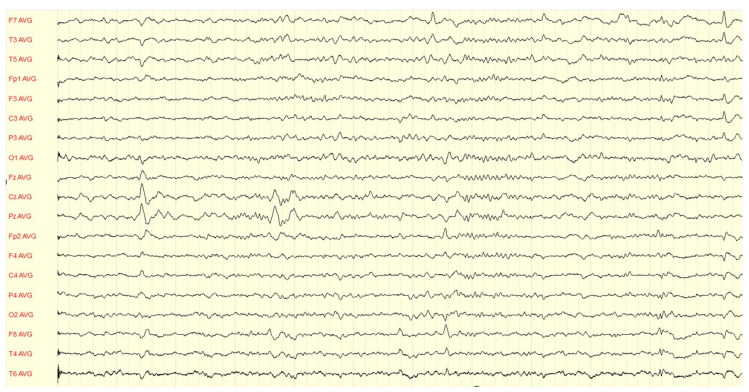
Sleep EEG findings: sharp waves in left temporal, fronto-centro-parietal, and right temporal areas.

Since the referral to our center in 2020, the patient has been followed up at the Department of Neurology of Lithuanian University of Health Sciences Kaunas Clinics. Routine biological tests, including biochemical tests and serum screening tests for infectious diseases, metabolic abnormalities, systemic autoimmune diseases, and thyroid function, were unremarkable. Brain MRI showed a left hippocampal malrotation and a small area of gliosis in the left amygdala without other remarkable findings (Figure 1). A brain 18F-2-deoxyglucose positron emission tomography (PET) scan showed no hypometabolic zones or other abnormalities. Several awake EEGs were recorded, showing interictal epileptiform discharges from the left and right temporal and frontotemporal regions (Figure 2 and Figure 3), and in an ictal EEG, a focal seizure was recorded originating from the left temporal lobe. Sleep EEG showed sharp waves in left temporal, fronto-centro-parietal, and right temporal areas (Figure 4).

In 2021, the patient underwent vagus nerve stimulator implantation, with a slightly positive effect on nocturnal focal to bilateral tonic–clonic seizures but no remarkable reduction in the frequency of focal seizures. Since the patient suffered from treatment-refractory epilepsy, in 2021, we decided to revisit the hypothesis of AAE. Testing revealed anti-GAD positivity in the serum: anti-GAD levels were 955 kU/L in February 2021 and >2000 kU/L in May and August 2021 (normal values in our laboratory are 5 kU/L); the anti-GAD65 titer was 1:320; and immunoblot testing showed a result of 2+ (medium, with the highest value being 3+). CSF analysis revealed 3 white blood cells per cubic millimeter, a slightly elevated protein concentration of 0.6 g/L, and a normal CSF/plasma glucose ratio of 0.62. Serum and CSF testing for antibodies against neuronal surface antigens were negative and serum testing for antibodies against intracellular neuronal antigens (e.g., anti-Hu, anti-Ri) other than anti-GAD was negative. Unfortunately, we were not able to perform testing for anti-GAD65 in the CSF. Routine blood tests, including complete blood count, basic metabolic testing, glycated hemoglobin, and thyroid function, were normal. Cancer screening was negative, and an Antibody Prevalence in Epilepsy and Encephalopathy (APE2) score was calculated with a result of 4 and in the presence of positive anti-GAD antibodies, conforming to definite autoimmune epilepsy according to the previous nomenclature. The patient had a family history of diabetes but did not have type 1 diabetes or any other explanation of positive anti-GAD antibodies; therefore, it was decided that in the context of refractory epilepsy, the most likely diagnosis was anti-GAD-positive autoimmune-associated epilepsy. 

Since there is no uniform consensus regarding the treatment of autoimmune-associated epilepsy, various treatment options were considered according to algorithms proposed in the literature. Due to the refusal by the patient and the risk of side-effects in the context of the pre-existing anxiety disorder, we decided not to choose corticosteroid therapy. A trial of plasma exchange was given in August 2021 with a total of 2750 mL of plasma removed during five sessions; however, no effect was seen one month later after the procedure. A course of intravenous immunoglobulin (IVIG) with a dose of 2 g/kg over 5 days was given in December 2021. Before the course of IVIG, the serum anti-GAD concentration was >2000 kU/L and the serum anti-GAD65 titer was 1:320 (December 2021). After the treatment, they decreased to 270 kU/L (March 2022) and 1:32 (May 2022), respectively. After the course of IVIG, the patient experienced no seizures for 2 weeks and then reported the disappearance of nocturnal focal to bilateral tonic–clonic seizures and a decreased focal impaired awareness seizure frequency, possibly attributable to the IVIG treatment; however, the precise change in frequency cannot be reported, since the patient did not keep a seizure diary. The anti-GAD concentration rebounded back to pre-treatment levels (>2000 kU/L) 6 months after treatment. During the short 2-week period after treatment with IVIG, when the patient experienced no seizures, she also reported a decrease in her anxiety level; however, it later returned to the baseline.

## 3. Review of Literature and Discussion

### 3.1. Etiology and Pathogenesis

Antibodies involved in the development of seizures can be classified into two groups: antibodies against neuronal surface antigens that are implicated in the development of acute symptomatic seizures secondary to autoimmune encephalitis and antibodies against intracellular antigens (anti-GAD and onconeural antibodies) that are found in cases of AAE [5].

Antibodies directed against neuronal surface antigens often target synaptic proteins and lead to the downregulation of receptors that alters synaptic transmission; therefore, they are directly pathogenic, whereas antibodies against intracellular antigens are not believed to be directly involved in the disruption of synaptic transmission and could be more of a reflection of an autoimmune process where not humoral but cellular immunity is the main factor leading to pathological changes [7]. Onconeural antibodies and anti-GAD trigger cytotoxic T-cell responses against the nervous tissue, as shown by several autopsy and biopsy series, which have revealed inflammatory infiltrates of cytotoxic T-cells around neurons in these cases [8]. These cytotoxic T cells may have different pathogenic mechanisms: T cells cause the release of perforin, which makes transmembrane pores in cells, leading to cell membrane swelling, rupture, and, eventually, cell necrosis. Moreover, T-cell-derived IFN-γ is responsible for the immediate loss of dendrites and synapses, and neuronal electrical activity is reduced below a critical threshold consequently [9].

In AAE, the predisposition to seizures is hypothesized to be a result of an ongoing immune response, structural damage to the brain parenchyma caused by it, or, most likely, a combination of both. The structural changes in AAE may not be visible on neuroimaging and may only be seen on a microscopic level [5]. Since, in autoimmune-associated epilepsy, it is the cellular immunity that predominates, and, after a longer disease course, there may be no ongoing immune response and it is only the functional or structural changes that predispose to seizures, immunotherapy is much less effective in comparison to acute symptomatic seizures secondary to autoimmune encephalitis. In the latter cases, there is a small subset of patients who continue to have seizures months later after the acute phase without any other signs of an ongoing immune process, possibly reflecting the residual structural damage that occurred in the initial phase of the disease. It is still a matter of debate among experts when the term AAE rather than acute symptomatic seizures secondary to autoimmune encephalitis should be used in these cases, with possible suggestions being 12 or 24 months of recurrent seizures after the acute phase of autoimmune encephalitis [10,11]. It should be mentioned that another cause of AAE is Rasmussen’s encephalitis in which unihemispheric chronic inflammation with recurrent seizures and resistance to anti-epileptic medications is present; however, a more detailed description of this disease is beyond the scope of this article [12].

Both antibodies against neuronal surface antigens and antibodies against intracellular antigens are associated with neoplastic processes with the association being stronger in the latter group. Tumor type and presence strongly depend on the type of autoantibodies: for example, in anti-Leucine-rich glioma-inactivated 1 (LGI1) encephalitis, there is only a 5–10% chance of cancer (usually thymoma), whereas in anti-Hu encephalomyelitis, only 15% do not have cancer, with the rest usually diagnosed with small cell lung carcinoma, and in anti-NMDA receptor encephalitis, approximately 50% of females older than 12 years have an ovarian teratoma [8,13,14].

### 3.2. Clinical Presentation, Workup, and Diagnosis

Acute symptomatic seizures secondary to autoimmune encephalitis occur in the acute phase of autoimmune encephalitis. The clinical presentation of these patients is very variable and includes combinations of seizures, prominent psychiatric manifestations, sleep and movement disorders, memory impairment, and autonomic instability among a few others [15]. Some autoimmune encephalitides have distinct clinical manifestations; for example, the most characteristic seizure type in anti-LGI1 encephalitis is faciobrachial dystonic seizures (FBDSs), which can occur as an isolated symptom at the beginning of the disease course [16,17]. Rarely, autoimmune encephalitis (especially anti-NMDA) can present only as an isolated psychiatric episode without any other neurological signs [18]. Contrary to autoimmune encephalitis, in the typical cases of AAE, there are no other neurological signs and drug-resistant epilepsy is most frequently the only clinical feature, with the exception being cases of persistent epilepsy after the acute phase of autoimmune encephalitis [12].

EEG findings are usually nonspecific and include slowing and epileptiform activity among others, and the only characteristic EEG pattern in autoimmune encephalitis is the extreme delta brush seen in approximately one-third of patients with anti-NMDAR encephalitis [19].

CSF examination is essential in the diagnosis of autoimmune encephalitis since approximately 60–80% of patients with autoimmune encephalitis have mild-to-moderate lymphocytic pleocytosis and some can have mild elevations of protein due to blood-cerebrospinal fluid barrier disruption [20,21]. In autoimmune encephalitis, oligoclonal bands are rarely found in the CSF (are absent in up to 60% of cases, especially in cases with anti-LGI1 [5]); however, in the case of their presence, an ongoing immune process should be highly suspected [21]. In AAE, the CSF may be completely normal or, more rarely, show mild pleocytosis (e.g., in anti-GAD65 autoimmune-associated epilepsy, the absence of pleocytosis was in 56% of cases) [21,22].

Neuroimaging is another essential step in the diagnosis. In acute symptomatic seizures secondary to autoimmune encephalitis, hyperintense signals on T2 FLAIR, which is highly restricted in multifocal grey and/or white matter and is compatible with demyelination or inflammation, are seen in brain MRI, and in cases of typical limbic encephalitis, the characteristic unilateral or bilateral T2 FLAIR restriction is present [20]. In AAE T2 or FLAIR, signal abnormalities can also be seen in the mesial temporal lobes or other brain regions, but they are usually much less prominent. In addition, in some cases, hippocampal sclerosis/atrophy can be present and, in others, the MRI may even be completely normal [21,23].

Finally, if autoimmune encephalitis or AAE is suspected, it is essential to send samples for antibody panels. Both CSF and serum can be tested and the sensitivity and specificity of testing in both samples depend on the type of antibody; for example, in anti-NMDAR encephalitis, CSF testing is more sensitive than serum testing [15]. However, 6–16% of patients with anti-LGI1 encephalitis and 13–14% of those with Contactin-associated protein-like 2 (CASPR2) antibodies are detected only in the serum, whereas both CSF and serum testing for anti-Gamma-Aminobutyric Acid type B (GABA_B_) receptors have equal sensitivity. In rare cases of autoimmune encephalitis, antibodies may be undetected both in serum and CSF, but in the context of a typical clinical presentation, neuroimaging findings, and CSF results, the diagnosis should be made nevertheless [21].

To aid the diagnostic process, a few diagnostic scales have been proposed, including the Antibody Prevalence in Epilepsy (APE) and the more recent Antibody Prevalence in Epilepsy and Encephalopathy (APE2) score [24,25]. A study that included patients with AAE or acute symptomatic seizures secondary to autoimmune encephalitis showed that an APE score ≥4 showed 77.9% specificity and 97.7% sensitivity to detect neural autoantibodies [24], and with its updated version, an APE2 score ≥4 was 99% sensitive and 93% specific for neural-specific antibodies [25]. In cases of recurrent seizures of unknown etiology, an APE2 <4 has been suggested to signify that an autoimmune etiology is unlikely, whereas in cases of APE2 ≥ 4, even in the absence of antibodies, an immunotherapy trial should be highly considered [26]. However, this scale was created when the term autoimmune epilepsy was still used and ILAE had not proposed the new definitions; therefore, it is problematic to apply in the current understanding of these disorders.

Even though, in the adult population, a genetic etiology of epilepsy is considered to be uncommon, it should be considered in certain atypical drug-resistant epilepsy cases. Shah et al. reported a case of a 20-year-old man who was initially treated for antibody-negative autoimmune encephalitis but eventually was diagnosed with POLG-related epilepsy after developing rapidly progressive valproate-induced hepatotoxicity, which is a distinctive feature of POLG mutations [27,28]. The authors of this case report suggested maintaining a careful differential in the case of suspected antibody-negative autoimmune encephalitis in young adults [27]. Since our patient did not have any other additional comorbid features seen in POLG-related epilepsy, did not develop hepatotoxicity after treatment with valproic acid, and was GAD-antibody-positive, we did not consider POLG-related epilepsy in our differential diagnosis.

The summary of differences between acute symptomatic seizures secondary to autoimmune encephalitis and autoimmune-associated epilepsy is provided in Table 1.

### 3.3. Treatment

Contrary to acute symptomatic seizures secondary to autoimmune encephalitis, AAE usually has a poor response to immunotherapy and is also resistant to anti-epileptic medications as well [5].

In AE, first-line treatment typically is IVIG, intravenous corticosteroids, or plasmapheresis. These treatments can be given in combination or individually. Rituximab and cyclophosphamide are the most commonly used second-line options when there is no response to first-line treatment. Treatment usually involves two phases—the induction therapy phase followed by a maintenance phase of a few weeks or months [15]. There is no universally accepted treatment scheme, and each center typically has its own practice particularities; however, therapeutic algorithms have been proposed in review articles [29].

If only antiseizure medications are used without addressing the underlying pathogenesis, the outcomes are not favorable, as was shown in one study with 103 patients who had anti-LGI1 autoimmune encephalitis and in whom cessation of FBDS occurred only in 10% of patients with AEDs alone and 51% showed cessation of FBDS after 30 days of immunotherapy [30].

In refractory cases, epilepsy surgery may be considered but is rarely effective [5]. There are less data on ketogenic diets, which can also be considered in autoimmune encephalitis and AAE cases. While on a ketogenic diet, adenosine levels in the brain increase, and this has anti-inflammatory effects [31]. In one small-case study, the ketogenic diet was used for patients with post-encephalitic and autoimmune-associated epilepsy, where 70% achieved more than a 50% seizure reduction, and one-third of patients became seizure-free [32].

Responsive neurostimulation therapy is a safe and promising treatment method for AAE. The research found an improvement in seizure frequency and duration reduction in 44% of patients [33]. However, it is not infrequent that AAE patients experience recurrent seizures despite all the available treatment options.

### 3.4. Autoimmune-Associated Epilepsy (GAD65)

There have been fewer than 200 cases of anti-GAD65 autoimmune-associated epilepsy reported in the literature; nonetheless, the incidence may be higher than was thought before due to low awareness of this disease and its diagnostic complexity [34].

There are two existing isoforms of glutamic acid decarboxylase—GAD65 and GAD67. GAD65 is responsible for GABA synthesis, the major inhibitory neurotransmitter in CNS. Isoforms of GAD65 are found in two main places: in CNS inhibitory (GABAergic) neurons and pancreatic islet β cells [35]. GAD has three functional domains: an amino (N)-terminal domain, a middle pyridoxal-5′-phosphate binding domain containing the active catalytic site of the enzyme, and a carboxy (C-terminal) domain. The antibodies against glutamic acid decarboxylase in different diseases have different binding targets. That is why antibodies against GAD65 can be associated with a wide spectrum of neurological syndromes, such as cerebellar ataxia, stiff-person syndrome, limbic encephalitis, and temporal lobe epilepsy. In patients with epilepsy, antibodies are more likely to react against the C-terminal, and in cases of limbic encephalitis—against the N-terminal of GAD [34]. In addition, anti-GAD65 can be detected in ~87% of diabetes mellitus type 1 patients but only 0.8% have anti-GAD > 2000 U/mL, so in cases of a suspected anti-GAD-associated neurological syndrome in a patient with diabetes, the diagnosis of an anti-GAD neurological syndrome must be made with caution, especially if the antibody levels are on the lower end [34,35,36].

Association with neoplastic processes is unusual in GAD autoimmunity and cancer screening is not routinely recommended unless there are additional factors: the clinical presentation is different from the typical GAD-associated epilepsy, the patient presents with a classic preneoplastic syndrome, or there is the presence of coexisting neuronal cell-surface antibodies [37].

Typically, anti-GAD65 AAE usually occurs in the second to third decade of life and is more common among females (70–80% of cases) and in those having other autoimmune diseases [34,35,36,38]. Seizures in the case of anti-GAD65 positivity can rarely present as part of an autoimmune encephalitis syndrome or, more frequently, as AAE; in both cases, seizures are usually of focal onset in temporal lobes [34]. Even though they are very rare, musicogenic reflex seizures may be more common in anti-GAD 65 AAE than in other epilepsies: one study showed that out of 1510 epilepsy patients (including 22 anti-GAD65), only three reported musicogenic reflex seizures and two of them had anti-GAD65 AAE, resulting in a prevalence of 9% [39,40].

There are no anti-GAD-AAE-specific changes in EEG or brain MRI and they conform to those described in previous sections. In most anti-GAD AAE cases, the CSF white blood cell count is normal but sometimes can be mildly elevated, and oligoclonal bands may be present in some cases as well [34]. If available, anti-GAD65 antibodies should be tested both in the serum and in the CSF since CSF positivity helps to assess the significance of anti-GAD65 antibodies that were positive in the serum [15]. Nonetheless, there is no association between the seizure frequency, severity, or duration of epilepsy and anti-GAD65 antibody titers. It is noticed that after initiation of immunotherapy, persistently high antibody titers are associated with poor clinical response [34].

The treatment of anti-GAD65 AAE involves three main aspects: AEDs, immunotherapy, and epilepsy surgery. No AED has been reported as superior in the treatment of anti-GAD65 AAE and multiple trials of various AEDs and their combinations are usually ineffective, and this was seen in our patient’s case as well. Patients usually do not have a good response to immunotherapy, which, interestingly, is also less effective in reducing seizure frequency in anti-GAD65 autoimmune encephalitis when compared to other autoimmune encephalitides [14], indicating a possible different pathophysiological mechanism that requires the development of a distinct approach. The most common immunotherapy approaches include IVIG, intravenous corticosteroids, or plasmapheresis as in other AAEs [35,36]. In a case series of 13 anti-GAD AAE patients, 5/11 patients had a ≥50% reduction in seizure frequency with corticosteroid treatment given as monthly pulses or continuously per os (median intervention duration—4 months with a range from one to seven months, median total dose of 19 g of methylprednisolone equivalent); however, severe side-effects occurred in 5 out of 11 patients: Cushing syndrome in three; sleep disorders, nervousness, diabetes mellitus, and psychosis in one each [35]. The same study showed a poor response to monthly IVIG (median total dose of 3 g/kg) and to apheresis with only 1/5 and 1/8 patients responding to treatment, respectively; with treatment, anti-GAD titers declined but did not become negative in most cases, suggesting an ongoing synthesis [35].

There are even less data regarding surgical treatment in anti-GAD65 AAE: one study showed that out of eight patients who underwent anterior temporal lobectomy, only two became at least almost seizure-free in the long term [41]. Another study reported the results of a selective amygdalohippocampectomy for three patients with anti-GAD65 epilepsy: post-surgery seizure frequency reduction was noticed but none of the patients achieved complete seizure freedom [35]. Bilateral hippocampal responsive neuromodulation system (RNS) treatment could potentially be useful as well: out of four anti-GAD65 AAE patients treated with the RNS System, three had a >50% clinical seizure reduction and one became clinically seizure-free following resective surgery informed by the RNS System data [42]. There are scarce data regarding vagus nerve stimulation: one case report showed that vagus nerve stimulation combined with two AEDs resulted in seizure freedom in a pediatric patient with anti-GAD65 AAE who had failed multiple AED trials, epilepsy surgery, and immunotherapy [41].

Our present report has demonstrated the complexity of the diagnosis and management of anti-GAD65 AAE. The delay from the diagnosis of epilepsy to the diagnosis of anti-GAD65 AAE was 8 years. Interestingly, in this case, levetiracetam and vagus nerve stimulations were partially effective in reducing the frequency of nocturnal focal to bilateral tonic–clonic seizures but had no effect on focal seizures with impaired awareness, whereas IVIG had a positive effect on both. In this case, the possibility of subsequent IVIG courses could be considered due to its partial efficacy, but since no clear recommendations exist and, in case series, even the same authors give treatment regimens with different doses and treatment durations, the clinical decision-making process is very complicated. In case a different immunotherapy was considered, as plasmapheresis had no effect on seizure frequency, glucocorticoids would remain the other possibility; however, as discussed previously, both their efficacy and risk of side-effects are the highest, and, in our opinion, the risk–benefit ratio in our case is too high given that the patient already suffers from a generalized anxiety disorder. The efficacy of natalizumab has been shown in one patient [35] but this does not seem like a possible treatment option with so little data and the elevated risk of progressive multifocal leukoencephalopathy. Taking all this into account, treatment with IVIG seems to be the best option in our case; however, multiple questions in our case remain unanswered: how many courses of IVIG, at what intervals and doses should it be given; should anti-GAD-raising antibody levels be used to guide IVIG course timing? The only data available at the moment are descriptive but with anti-GAD65 AAE being a rare complex disease, the conduction of randomized controlled trials would be challenging; therefore, the creation of a multinational database could be of great value.

## 4. Conclusions

This case represents anti-GAD65 AAE and highlights the complexity of its treatment and diagnosis. Even though AAE seems to be more common than previously thought, the diagnostic delay can extend up to a decade or more, and even after a correct diagnosis, the treatment of AAE remains a great challenge. AAE diagnosis should be considered for patients with treatment-resistant epilepsy who usually have none or only mild, other neurological signs or MRI or CSF changes. In order to distinguish acute symptomatic seizures secondary to autoimmune encephalitis from AAE, one should take into account the disease course, CSF examination results, and neuroimaging findings. Extensive research and data analysis are necessary in order to create clear guidelines for the diagnosis and treatment of AAE.

## Figures and Tables

**Table 1:** Summary of differences between acute symptomatic seizures secondary to autoimmune encephalitis and autoimmune-associated epilepsy.

	Acute Symptomatic Seizures Secondary to Autoimmune Encephalitis	Autoimmune-Associated Epilepsy
Antibodies involved	Antibodies against neuronal surface antigens (e.g., NMDAR, LGI1, GABABR, GABAAR).	Antibodies against intracellular antigens (anti-GAD and onconeural antibodies).
Pathogenesis	Antibodies often target synaptic proteins that lead to the downregulation of receptors, which alters synaptic transmission.	Cellular immunity (T-cell-mediated brain inflammation) is the main factor leading to pathological changes. Ongoing immune response, structural damage to the brain parenchyma, or combination of both. Recurrent seizures 12 or 24 months after the acute phase of autoimmune encephalitis. Rasmussen’s encephalitis.
Clinical presentation	Very variable and includes combinations of seizures, prominent psychiatric manifestations, sleep and movement disorders, memory impairment, and autonomic instability. MRI is often abnormal.	There are no other neurological signs and drug-resistant epilepsy is most frequently the only clinical feature. MRI is usually unremarkable.
Treatment	Usually, there is a good response to immunotherapy, which is the mainstay of treatment. Antiseizure medications.	Poor response to immunotherapy and resistance to antiseizure medications. Epilepsy surgery is rarely effective.

## Data Availability

Not applicable.
